# RNA3DB: A dataset for training and benchmarking deep learning models for RNA structure prediction

**DOI:** 10.1101/2024.01.30.578025

**Published:** 2024-02-02

**Authors:** Marcell Szikszai, Marcin Magnus, Siddhant Sanghi, Sachin Kadyan, Nazim Bouatta, Elena Rivas

**Affiliations:** a Department of Molecular and Cellular Biology, Harvard University, Cambridge, 02138, MA, USA; b Department of Systems Biology, Columbia University, New York, 10027, NY, USA; c College of Biological Sciences, UC Davis, Davis, 95616, CA, USA; d Laboratory of Systems Pharmacology, Harvard Medical School, Boston, 02115, MA, USA

## Abstract

With advances in protein structure prediction thanks to deep learning models like AlphaFold, RNA structure prediction has recently received increased attention from deep learning researchers. RNAs introduce substantial challenges due to the sparser availability and lower structural diversity of the experimentally resolved RNA structures in comparison to protein structures. These challenges are often poorly addressed by the existing literature, many of which report inflated performance due to using training and testing sets with significant structural overlap. Further, the most recent Critical Assessment of Structure Prediction (CASP15) has shown that deep learning models for RNA structure are currently outperformed by traditional methods.

In this paper we present RNA3DB, a dataset of structured RNAs, derived from the Protein Data Bank (PDB), that is designed for training and benchmarking deep learning models. Our dataset clusters RNA 3D chains into distinct groups that are non-redundant both with regard to sequence as well as structure, providing a robust way of dividing training, validation, and testing sets. For the PDB RNA chains as of 2024-01-10, RNA3DB produces 118 independent components with a total of 1,645 distinct RNA sequences with 21,005 reported crystal structures, representing 216 different Rfam structural families. A potential split consists of a training set of 1,152 RNA sequences, with 9,832 experimentally determined structures that belong to 169 distinct RNA structural Rfam families (at an E-value of 10^−3^), and a test set of 493 RNA sequences with 1,344 structures that belong to 47 structural Rfam families. This split guarantees that all test RNA chains are distinct by sequence and structure from those in the training set. We provide the methodology along with the source-code, with the goal of creating a reproducible and customizable tool for RNA structure prediction.

## Graphical Abstract

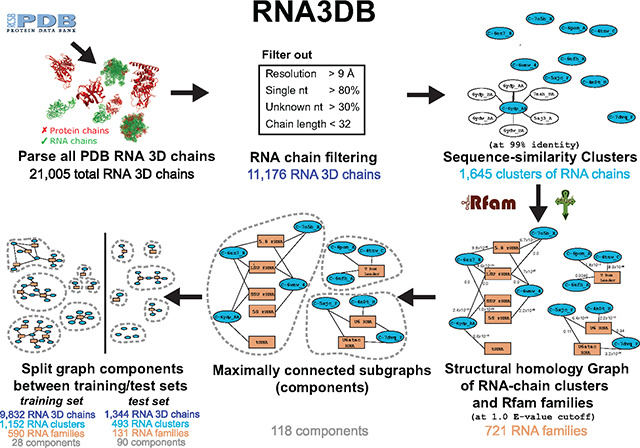

## 1. Introduction

Since DeepMind introduced AlphaFold [1] at CASP13 in 2018, there has been growing interest in applying deep learning to problems in structural biology [2]. When AlphaFold2 improved on its predecessor’s already impressive results in 2020, many jumped to claim that protein structure determination was “solved” [3].

Naturally it didn’t take long for lessons from AlphaFold to start being adapted for RNA [4, 5, 6, 7, 8, 9, 10, 11, 12], with some papers immediately reporting impressive results both for RNA secondary and tertiary structure prediction. Certainly at first glance, RNA structure appears to be in some ways analogous to protein structure. In both cases, the one-dimensional polymer sequences fold into three-dimensional conformations which are strongly tied to the molecule’s function, and strongly dependent on the molecule’s sequence.

Despite this, most RNA biologists do not consider deep learning methods to be state-of-the-art for RNA structure prediction [13] for secondary or tertiary structures. Publications began pointing out issues with generalization to unseen sequences in deep learning methods for secondary structure prediction in 2022 [14, 15, 16, 17], although this overfitting behavior was already well-known for probabilistic and Markov random field models trained on structural RNA data since 2012 [18]. With the increased interest in RNA, partly attributed to AlphaFold’s success, and partly due to the rise of RNA-based therapeutics [19], CASP15 added 12 RNA-only targets to the competition [20, 21], where all deep learning methods performed comparatively poorly to traditional tools [22]. Since CASP15, several other publications have attempted to apply deep learning to the RNA problem including DeepMind [23], many of whom report impressive results [22], but often without addressing the concerns regarding generalization.

**Figure 1:**
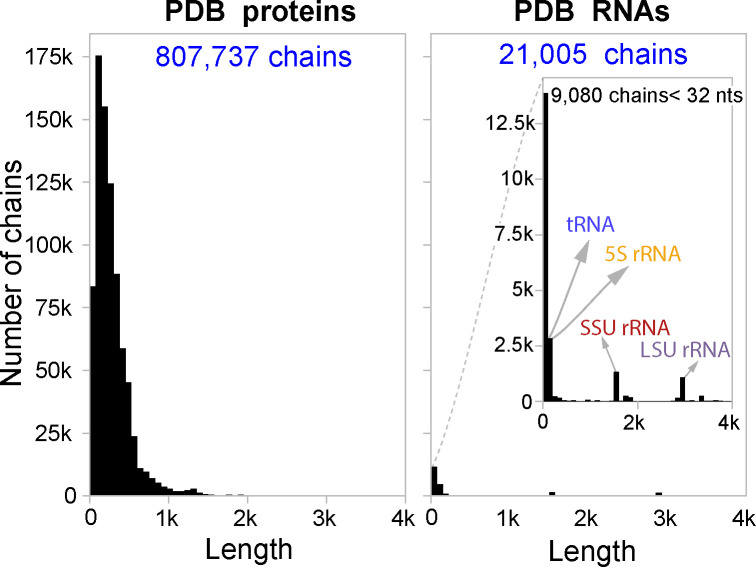
Comparison of length distributions of chains in the PDB for proteins and RNAs at the same y-axis scale. The inset plot shows a zoomed in version of the RNA histogram for visibility.

There is a widespread belief in deep learning that the quantity and quality of datasets is one of the most influential aspects towards a model’s performance. The number of available proteins is significantly higher than RNAs in the Protein Data Bank (PDB). Figure 1 shows a comparison between the number of protein and RNA experimentally determined structures deposited in PDB (named chains). After filtering (see Section 2.2), PDB contains nearly 70 times more available protein chains than RNA chains.

**Figure 2:**
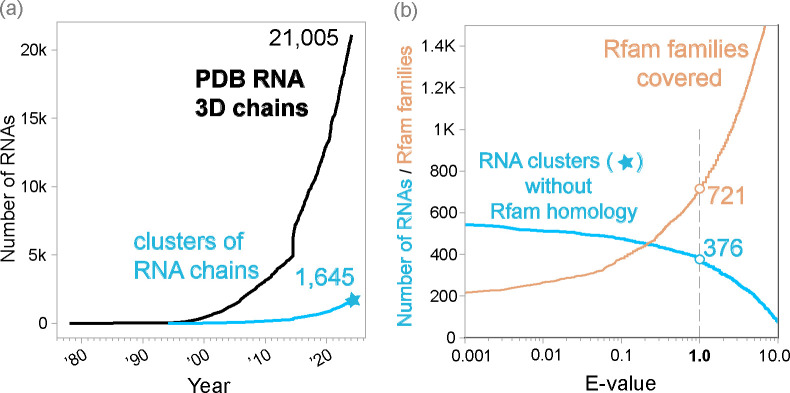
Panel **(a)** shows: in black the cumulative number of PDB chains that contain at least one RNA residue based on the _chem_comp.type data item by year; and in blue the cumulative number of distinct RNA sequences (sequence identity threshold of 99%) represented in the PDB RNA 3D chains. Panel **(b)** shows in blue the number of distinct RNA sequences in the PDB as of 2024-01-10 (blue star in (a)) without significant homology to any Rfam structural RNA family at different E-value thresholds (as calculated by the Infernal method cmsearch). Panel **(b)** shows in orange the diversity of Rfam RNA structures covered by the RNA chains at different E-value cutoffs.

With this in mind, it is perhaps no surprise that deep learning for RNA is less successful than for proteins. While other problems are potentially solvable engineering challenges, the quantity and diversity of available data cannot be easily increased. Fortunately, the number of novel structures uploaded to the PDB each years appears to be increasing (Figure 2), albeit still very far away from proteins. It is important to note that the PDB, while comprehensive and consistently used by the structural community, is just a repository, and not a dedicated tool to provide deep learning models with a curated dataset, leading to several challenges. For instance, many of the RNAs included in the PDB are short fragments of longer RNAs, and many of the entries contain the same or effectively the same RNA sequences repeatedly.

RNA3DB is a dataset based on all PDB RNA 3D structures developed for addressing the aforementioned concerns, especially those regarding generalization, particularly for training and benchmarking deep learning models. The methodology behind RNA3DB clusters the RNA 3D structures into distinct groups that are non-redundant both with regard to sequence as well as structure, providing a robust way of dividing training, validation, and testing sets.

## 2. Materials and Methods

Our methodology for building RNA3DB can be broken down into four main steps: parsing, filtering, clustering, and splitting. During the parsing step, all PDB chains are processed to identify potential RNAs. Filtering then removes sequences that are unsuitable for deep learning for various reasons, such as length or resolution. Next, clustering assigns PDB chains into two hierarchies of groups based on sequence similarity and then structural similarity. As part of clustering, a graph of structural homology between RNA chains and RNA structural families allows us to calculate structurally dissimilar groups or graph components. Finally, splitting assigns PDB chains into training and testing sets that are non-redundant both in terms of sequence and structure.

### 2.1. Parsing

Our method starts with careful parsing of all entries in the PDB to identify any potential RNA structures. This is done by downloading a copy of all PDB entries in PDBx/mmCIF format. To avoid reliance on author labeling, we scan all chains for the chem_comp data category, which is found in practically all PDBx/mmCIF files [24]. During our first pass, we accept any chains with at least one residue containing “RNA” in its _chem_comp.type data item. Any non-RNA residues, such as amino acids, are treated as “unknown” residues at this stage.

An important parsing issue is that of RNA residue modifications such as pseudouridylations. The number of modified residues in an RNA chain can be large. As an example, for the tRNA structure PDB:1EHZ [25, 26] 2 out of the 72 residues are pseudouridines, and 10 are other modified residues. A naive parsing method usually reports these modifications as “unknown” residues.

The RNA3DB method systematically converts all modified nucleic acids to their closest one-letter symbols. We extract all three-letter symbol conversions of nucleic acids (and proteins for possible future use) from the Chemical Component Dictionary, which is an “external reference file describing all residue and small molecule components found in PDB entries” [27]. In addition to naively converting three-letter codes, we also recursively parse parent components from _chem_comp.mon_nstd_parent_comp_id to maximize the number of extracted modifications. Any three-letter codes that cannot be converted (including stray amino acids) are parsed as “unknown” residues. Our method is comprehensive method, and is able to identify up to 582 different nucleic acid modifications.

### 2.2. Filtering

Next, the filtering step aims to remove chains that are not informative for training deep learning models. By default, RNA3DB considers four filtering categories: sequence length, structural resolution, fraction of individual nucleotides in the sequence, and fraction of “unknown” residues.

Chains shorter than 32 residues are removed during this step. In many cases, there is not much information about the structure in only a few nucleotides, and these sequences can be largely ignored. However, it should be noted that this is not always true. There are also some cases where some of these shorter chains are potentially informative fragments of longer RNAs (for instance, chain PDB: 354D_A^1^ [28, 29] is a crystal structure exclusively of the 12 nucleotide long Loop E of 5S rRNA). Since these short motifs are difficult to classify into families–as due to their short length they are hard to distinguish from random sequences–we opt to remove these from the dataset as it may lead to data leakage. We experimented with methods to attempt to both identify and keep these fragments, but were unable to systematically avoid scenarios where known fragments of longer chains overlapped between training and testing sets.

Chains with structural resolution higher than 9 Å are removed, as they are considered to have too low confidence to be useful to determine atom positions. This is the same threshold that AlphaFold2 uses [1]. By default RNA3DB also excludes any structures resolved with nuclear magnetic resonance spectroscopy (NMR), as NMR does not provide well-defined resolution values. However this only excludes a relatively small number (177) of chains that would otherwise not be removed. An optional flag exists within RNA3DB’s parser that interprets the resolution of NMR structures as 0.0 Å, which allows these structures to be included if desired.

The sequence nucleotide composition is also considered to avoid repetitive sequences with low information content. By default, we remove any sequence where a single nucleotide makes up more than 80% of residues, like AlphaFold2 [1].

Finally, we also remove any sequence where more than 30% of the residues are “unknown”. This removes chains that do not provide sufficient information in their sequences, but also acts as a filter that removes any special cases where a non-RNA polymer is parsed as one because the sequence may contain “RNA” in its _chem_comp.type (see Section 2.1).

### 2.3. Clustering

Clustering is divided into three distinct steps: sequence-based clustering, structure-based clustering, and identification of connected subgraphs. These steps create a “hierarchy” in RNA3DB, starting with RNA chains, to sequence-based “clusters”, followed by structure-based independent “components”. Each cluster guarantees that its sequences are not identical (or near-identical) to any other clusters’, while each component guarantees that it shares no structural homology to the same RNA families as any other component.

First, MMseqs2 [30] is used to cluster all 3D RNA chains by 99% sequence identity. Many of the RNA chains in the PDB have identical or near-identical sequences, and the purpose of this step is to group them together in clusters. All RNA chains in one cluster have almost identical RNA sequences, but each chain is associated to a different experimentally determined structure. A given cluster may contain RNA chains identical to a region of a larger chain. RNA3DB selects the longest RNA chain as representative of the cluster, which is named as “cluster <chain_name>”. We observe (Figure 2a) that the number of RNA-chain sequence-based clusters is about a tenth that of the number of actual RNA chains.

Second, Infernal [31] is used to run an RNA homology search against all Rfam families [32]. We find it convenient to have this information for all existing sequences in the PDB, however, this step can be restricted to only the unique sequences after sequence-based clustering for the purpose of building RNA3DB. We use a two-pass approach to maximize the number of chains for which we get at least one hit, regardless of significance. The first pass uses default Infernal parameters. The second pass re-runs the search on sequences without any hits, except with all filters turned off. The purpose of this second pass is to increase sensitivity, but it is generally very slow.

RNA3DB uses these comprehensive homology searches to create structurally dissimilar groups of RNA structures. A graph is constructed as follows: let each RNA-chain cluster be a node in the graph, and all Rfam families also be nodes in the graph. Edges are undirected and unweighted between chain and family nodes and are present when some E-value threshold is met. By default we use a generous E-value threshold of 1.00, since we want to eliminate the possibility of missing any potential homology, with false positives being an acceptable trade-off.

Finally, the RNA3DB method performs a depth-first search on the graph described above to identify all maximally connected subgraphs, named components. This way, we guarantee that all components share no homology to the same families. These components are then ranked by size (i.e. number of unique chain clusters in the component) with the exception of component #0, which includes all chain-nodes without edges to any RNA family nodes.

We can motivate building these structurally dissimilar components via some clear examples. Take a 55S mammalian mitochondrial ribosome like PDB:6YDP_AA [33, 34]. Infernal finds significant hits (at an E-value threshold of 10^−9^) to both LSU and SSU rRNA families, as well as several other hits to tRNAs within the sequence. While rRNA and tRNA families have no homology, this chain must be used in the same training/testing set with other individual tRNA and rRNA chains to avoid inadvertently leaking structural information from the training set into the testing set.

It may be surprising to find out that for 376 chains in the PDB, i.e. those in component #0, we are unable to find homology to any Rfam family at an E-value threshold of 1.0. Many of these chains are synthetically designed structures, messenger RNA fragments crystallized as part of translational complexes, or in some cases structural fragments that are too short, even above 32 residues, to classify at the desired threshold.

### 2.4. Splitting

This is the final step, which assigns the clustered chains into training and testing sets. Since any two components of the graph from the clustering step (Section 2.3) are completely non-redundant, the components can safely be placed arbitrarily into any set without data leakage.

RNA3DB provides an algorithm for dividing the graph components into training/test sets. The algorithm simply assigns components with the largest number of unique sequences into the training set until a specified training set split percentage is met. By default, we recommend a split of 70–30, or in other words, include the largest components in the training set (with the exception of component #0) until at least 70% of the data is in the training set.

We recommend using component #0 for testing rather than training to minimize the chance of data leakage, as well as following structure prediction benchmarking best practices, particularly with regards to reporting results per family instead of an overall average [35]. Alternatively, RNA3DB gives the option to ignore this component #0 all together.

It should be noted that this splitting step can also be done manually with relative ease. Using default parameters, RNA3DB finds 118 graph components, which is a manageable set for manual inspection.

## 3. Results

Among the most important observations from our dataset is that approximately 1 in 10 RNA PDB chains are either redundant in sequence or too short to be usable by deep learning methods (Figure 2 (a)). Despite this, it is clear that the number of novel RNAs uploaded has increased over recent years.

The RNA3DB parser finds 21,005 RNAs in the PDB as of 2024-01-10. The length filter removes 9,080 chains, while the resolution filter removes 1,540 chains. We find 1,294 sequences dominated by one nucleotide, and 177 that have too many unknown residues to keep. Note that a single chain may be rejected by more than one filter. After filtering 11,176 RNA chains remain, and 9,829 chains are rejected.

Next, the RNA3DB method produces 1,645 sequence-similarity clusters (at 99% identity) of RNA chains. The largest cluster with 629 RNA chains is the *Thermus thermophilus HB8* 70S ribosome. The median number of chains per cluster is 2.0.

Then RNA3DB proceeds to make a graph which adds 721 Rfam family nodes (at an E-value threshold of 1.0) to the 1,645 RNA-chain cluster nodes (Figure 2b). The RNA3DB resulting graph has a total of 3,994 edges. The tRNA (RF00005) family has the largest number of edges (307), and the median number of edges per RNA family node is 2.0. The cluster with the largest number of edges (43) is 6ydp_AA (the 55S mammalian mitochondrial ribosome), and the median number of edges per cluster node is 2.0.

Finally, the RNA3DB method produces 118 non-redundant components. The largest component, component #1, includes 119 RNA families and 935 RNA-chain clusters. More than half of the components include one single RNA family and one single RNA cluster of chains.

The component #0 set comprises all RNA chains without hit to any Rfam family. At an E-value threshold of 1.0, component #0 contains 376 RNA clusters and 979 actual RNA chains (Figure 2b), and it includes synthetic RNAs as well as small messenger RNA sequences crystillized as part of larger complexes.

The RNA3DB dataset provides a training/test dataset split described in Table 1. The training/testing mmCIF files for the chains in both sets (after converting modified residues) can be downloaded directly from https://github.com/marcellszi/rna3db/XXX.

## 4. Implementation

The database and code for RNA3DB can be found at: https://github.com/marcellszi/rna3db along with documentation on the Github repository’s Wiki at https://github.com/marcellszi/rna3db/wiki.

For the RNA3DB dataset provided with this manuscript, we used Rfam version 14.10, Infernal version 1.1.4, and we included all RNA chains from PDB as of 2024-01-10. The whole process, with the exception of the homology search, can be run on a 10 core Apple M2 Pro in under 2 minutes. The homology search took 110 hours on a single Intel Xeon Platinum 8358 processor with 32 cores.

The RNA3DB method can be customized to build specialized train/test datasets. For instance, filtering parameters (such as the minimal length) can be modified. Different train/test independent splits can be created depending on the specified parameters, which are documented in the Github Wiki.

## 5. Discussion

The development of a standardized dataset of RNA structures specifically targeting deep learning, is extremely valuable. Here, we introduce RNA3DB a method to obtain comprehensive information about structured RNAs in the PDB in a format that organizes the RNA chains by their structural homology. RNA3DB builds datasets of maximally connected RNA chains with information on the actual structures represented in each group.

RNA3DB exhaustively parses all modified residues present in the experimental RNA chains, and the method is customizable in a number of ways such as minimal length or sequence complexity. RNA3DB makes apparent the reduced amount of structural RNA data present in the PDB when compared to that of proteins, and the limited set of distinct 3D RNA structures that it represents.

The sparsity of data alone could be responsible for a poor performance of deep learning methods dependent on millions of parameters to predict RNA structure, as it was seen at CASP15 [20, 21] for 3D structure prediction, as well as for 2D structure prediction [14, 15, 16, 17]. Other factors possibly handicapping the prediction of RNA structure are the more complex RNA backbone geometry that involves more atoms and degrees of freedom, as well as global nature of RNA secondary structure. The global nature of the secondary structure, unlike with proteins, cannot be inferred locally from contiguous residues, and it substantially informs the 3D structure.

Nevertheless, the sparsity of data deserves prioritized consideration. Would a method trained with very limited data be able to generalize to describe not seen before RNA structures? The realm of image data analysis seems to suggest that deep learning methods are able to generalize even when the amount of training data can just be memorized by the method [36, 37]. The RNA3DB method and its final outcome the RNA3DB dataset is a comprehensive classification of structurally dissimilar RNA experimentally-determined structures. We hope that this tool will provide the RNA structure modelling community an effective tool to investigate this question under different settings with rigor.

## Supplementary Material

Supplement 1

## Figures and Tables

**Table 1:** Hierarchical table of a training/testing split. A partial representation of all tiers of hierarchy (components, sequences, chains and RNA families) for both training and testing sets is shown. RNA3DB uses by default an Infernal E-value cutoff of 1.0 to generate the graph.

RNA3DB	Graph components	RNA sequences	RNA chains	RNA families E-val < 10^−3^	RNA families E-val < 1.0	Description
Training	28	1,152	9,832	169	590	
	component #1	935	9,037	119	508	rRNA (LSU,SSU,5.8,5S), tRNA, tmRNA, U1, etc,
	component #2	24	24	24	24	Purine/2dG-II riboswitches
	component #3	22	82	2	5	tracrRNA, CRISPR-DR22
	component #4	18	69	2	3	Group I intron
						:
	component #28	3	11	1	1	Bacteriophage pRNA
Testing	90	493	1,344	47	131	
	component #0	376	979	0	0	synthetic RNAs, mRNAs, etc.
	component #29	3	12	1	1	THF riboswitch
	component #30	3	12	1	1	ZMP-ZTP riboswitch
	:					:
	component #116	1	2	0	1	L25-Gammaproteobacteria ribosomal protein leader
	component #117	1	1	0	1	mir-3135 microRNA precursor
Total	118	1,645	11,176	216	721	

## Footnotes

1 Chain naming is done via author labeling.
