# Supplementary figures and images for: RNA3DB: A structurally-dissimilar dataset split for training and benchmarking deep learning models for RNA structure prediction
Source: bioRxiv. 2024 Mar 11:2024.01.30.578025. Preprint. [Version 3] doi: 10.1101/2024.01.30.578025 (PMC10862857; doi:10.1101/2024.01.30.578025)

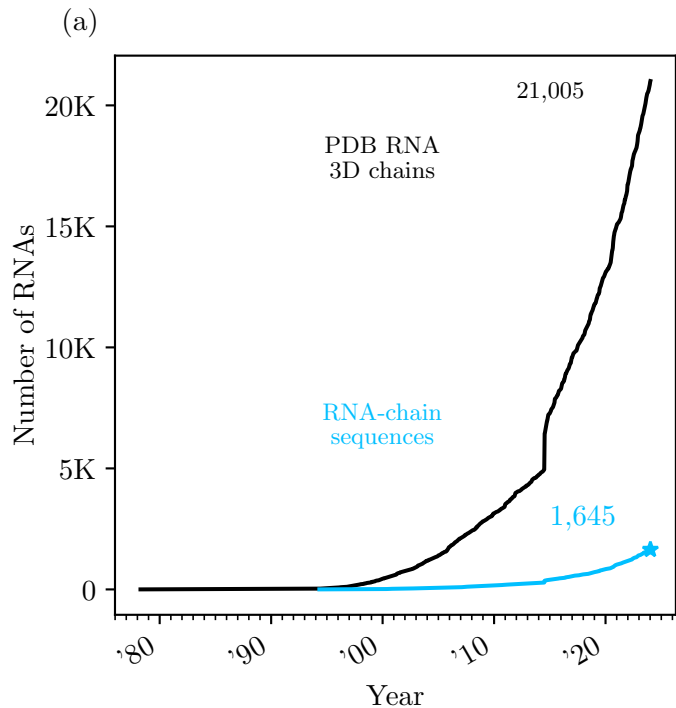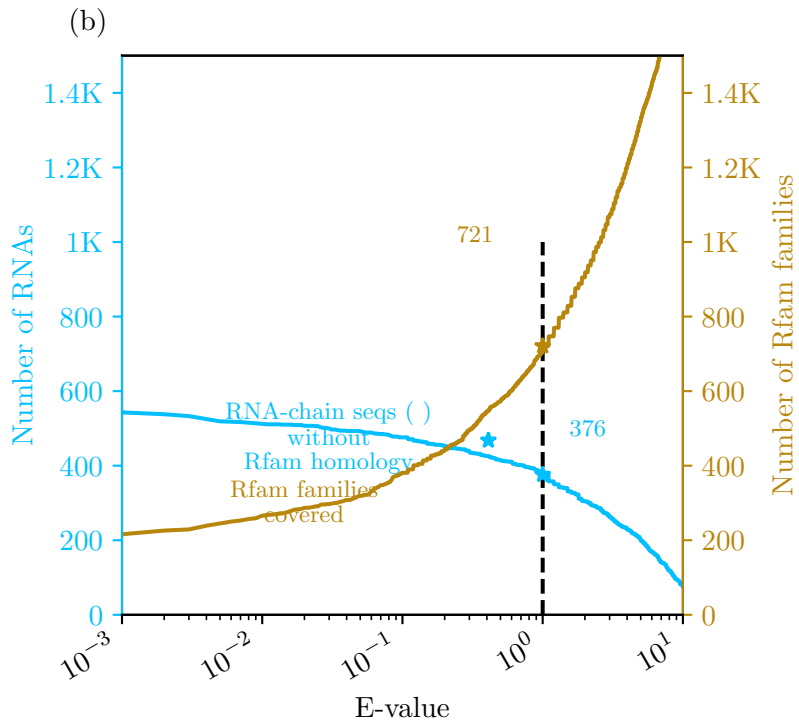

Supplement: Supplement 1 [file media-1.gz › supplemental_material/Figure2/Figure_2.pdf]
