# Supplementary material for: RNA3DB: A structurally-dissimilar dataset split for training and benchmarking deep learning models for RNA structure prediction
Source: bioRxiv. 2024 Mar 11:2024.01.30.578025. Preprint. [Version 3] doi: 10.1101/2024.01.30.578025 (PMC10862857; doi:10.1101/2024.01.30.578025)
Supplement: Supplement 2 [file media-2.pdf]

Supplementary Information

RNA3DB: A dataset for training and benchmarking deep learning models for RNA structure prediction

Marcell Szikszai[a], Marcin Magnus[a], Siddhant Sanghi[b,c], Sachin Kadyan[b], Nazim Bouatta[d], Elena Rivas[a]

[a] *Department of Molecular and Cellular Biology, Harvard University, Cambridge, 02138, MA, USA*
[b] *Department of Systems Biology, Columbia University, New York, 10027, NY, USA*
[c] *College of Biological Sciences, UC Davis, Davis, 95616, CA, USA*
[d] *Laboratory of Systems Pharmacology, Harvard Medical School, Boston, 02115, MA, USA*

Contents

1 Table of PDB RNA chains with homology to multiple Rfam Clans 2

2 Table of PDB RNA chains with homology to multiple Rfam families that do not share a common Rfam Clan 3

Preprint submitted to J. Mol. Biol.

1. Table of PDB RNA chains with homology to multiple Rfam Clans

Table S1: PDB RNA chain cluster representatives (at 99% sequence similarity) with homology to multiple Rfam Clans. We provide all Infernal `cmscan` hits at an E-value threshold of 10^{-3}.

RNA chain	Start	End	Rfam accession	Name	Clan accesion	Clan name	E-value
7am2_1	1,904	2,470	RF02546	LSU_trypano_mito	CL00112	LSU	1.4e-138
7am2_1	2,491	3,117	RF02545	SSU_trypano_mito	CL00111	SSU	4e-129
6hrm_1	1,507	4,403	RF02541	LSU_rRNA_bacteria	CL00112	LSU	0.0
6hrm_1	1,506	4,411	RF02540	LSU_rRNA_archaea	CL00112	LSU	0.0
6hrm_1	1	1,528	RF00177	SSU_rRNA_bacteria	CL00111	SSU	0.0
6hrm_1	1,666	4,414	RF02543	LSU_rRNA_eukarya	CL00112	LSU	0.0
6hrm_1	6	1,460	RF01959	SSU_rRNA_archaea	CL00111	SSU	3.5e-274
6hrm_1	6	1,457	RF02542	SSU_rRNA_microsporidia	CL00111	SSU	8.3e-199
6hrm_1	6	1,522	RF01960	SSU_rRNA_eukarya	CL00111	SSU	8.4e-181
6hrm_1	1,519	1,672	RF00002	5_8S_rRNA	CL00112	LSU	2.5e-07
6hrm_1	4,372	4,445	RF00177	SSU_rRNA_bacteria	CL00111	SSU	0.00072
7aor_2	2,020	2,579	RF02546	LSU_trypano_mito	CL00112	LSU	9.7e-138
7aor_2	2,589	3,210	RF02545	SSU_trypano_mito	CL00111	SSU	4.5e-120
6wnw_4	1	3,031	RF02541	LSU_rRNA_bacteria	CL00112	LSU	0.0
6wnw_4	1	3,031	RF02540	LSU_rRNA_archaea	CL00112	LSU	0.0
6wnw_4	160	3,021	RF02543	LSU_rRNA_eukarya	CL00112	LSU	0.0
6wnw_4	1,069	1,185	RF00001	5S_rRNA	CL00113	5S_rRNA	2.2e-08
6wnw_4	13	166	RF00002	5_8S_rRNA	CL00112	LSU	1.7e-07
7aih_1	2,400	2,963	RF02546	LSU_trypano_mito	CL00112	LSU	8.6e-140
7aih_1	2,984	3,610	RF02545	SSU_trypano_mito	CL00111	SSU	3.5e-130
6ydp_AA	1,176	2,737	RF02541	LSU_rRNA_bacteria	CL00112	LSU	2.5e-131
6ydp_AA	1,173	2,738	RF02540	LSU_rRNA_archaea	CL00112	LSU	9.8e-92
6ydp_AA	66	1,037	RF00177	SSU_rRNA_bacteria	CL00111	SSU	1e-80
6ydp_AA	71	1,035	RF01959	SSU_rRNA_archaea	CL00111	SSU	2.2e-56
6ydp_AA	71	1,032	RF02542	SSU_rRNA_microsporidia	CL00111	SSU	3.4e-46
6ydp_AA	2,141	2,541	RF02543	LSU_rRNA_eukarya	CL00112	LSU	8e-33
6ydp_AA	1,915	2,080	RF02543	LSU_rRNA_eukarya	CL00112	LSU	5.2e-12
6ydp_AA	2,669	2,743	RF00005	tRNA	CL00001	tRNA	6.6e-10
6ydp_AA	5,333	5,268	RF00005	tRNA	CL00001	tRNA	1.2e-09
6ydp_AA	3,701	3,769	RF00005	tRNA	CL00001	tRNA	1.6e-09
6ydp_AA	3,839	3,767	RF00005	tRNA	CL00001	tRNA	1.4e-08
6ydp_AA	9,408	9,476	RF00005	tRNA	CL00001	tRNA	2.3e-07
6ydp_AA	11,561	11,629	RF00005	tRNA	CL00001	tRNA	5.6e-07
6ydp_AA	14,159	14,091	RF00005	tRNA	CL00001	tRNA	1.9e-06
6ydp_AA	5,094	5,027	RF00005	tRNA	CL00001	tRNA	1e-05
6ydp_AA	5,170	5,096	RF00005	tRNA	CL00001	tRNA	1.6e-05
6ydp_AA	1	70	RF00005	tRNA	CL00001	tRNA	1.7e-05
6ydp_AA	3,841	3,910	RF00005	tRNA	CL00001	tRNA	0.00028
6ydp_AA	6,951	6,883	RF00005	tRNA	CL00001	tRNA	0.00041
7lyg_A	32	102	RF02340	DENV_SLA	CL00129	Flavivirus-5UTR	2.8e-10
7lyg_A	1	139	RF00005	tRNA	CL00001	tRNA	1e-09
6uz7_8	1	1,507	RF01960	SSU_rRNA_eukarya	CL00111	SSU	0.0
6uz7_8	1	1,507	RF02542	SSU_rRNA_microsporidia	CL00111	SSU	3.3e-238
6uz7_8	1	1,510	RF01959	SSU_rRNA_archaea	CL00111	SSU	1.6e-180
6uz7_8	1	1,512	RF00177	SSU_rRNA_bacteria	CL00111	SSU	1.2e-164
6uz7_8	2,140	2,825	RF02543	LSU_rRNA_eukarya	CL00112	LSU	4.6e-141
6uz7_8	1,724	2,825	RF02541	LSU_rRNA_bacteria	CL00112	LSU	8.6e-84
6uz7_8	1,975	2,825	RF02540	LSU_rRNA_archaea	CL00112	LSU	2.1e-71
6uz7_8	1,736	1,889	RF00002	5_8S_rRNA	CL00112	LSU	9.9e-45
7lyf_A	31	100	RF02340	DENV_SLA	CL00129	Flavivirus-5UTR	6.6e-16
7lyf_A	31	139	RF03546	Flavivirus-5UTR	CL00129	Flavivirus-5UTR	8.8e-11
7lyf_A	1	136	RF00005	tRNA	CL00001	tRNA	4.6e-10
8dfv_E	1	57	RF00027	let-7	CL00148	let-7	3.6e-09
8dfv_E	57	2	RF00027	let-7	CL00148	let-7	8.2e-09
8dfv_E	56	1	RF04289	mir-3596	CL00148	let-7	1.1e-05
8dfv_E	3	57	RF04289	mir-3596	CL00148	let-7	4.1e-05
8dfv_E	58	1	RF04292	mir-379	CL00149	mir-154	0.00024

2. Table of PDB RNA chains with homology to multiple Rfam families that do not share a common Rfam Clan

Table S2: PDB RNA chain cluster representatives (at 99% sequence similarity) with homology to multiple Rfam families that do not share a common Clan. We provide all Infernal `cmscan` hits at an E-value threshold of 10^{-3}.

RNA chain	Start	End	Rfam accession	Name	Clan accesion	Clan name	E-value
7am2_1	1,904	2,470	RF02546	LSU_trypano_mito	CL00112	LSU	1.4e-138
7am2_1	2,491	3,117	RF02545	SSU_trypano_mito	CL00111	SSU	4e-129
1ser_T	1	91	RF00005	tRNA	CL00001	tRNA	5.4e-15
1ser_T	1	90	RF01852	tRNA-Sec	CL00001	tRNA	3.2e-08
1ser_T	1	94	RF02223	sX4			4.6e-06
7nww_z	1	85	RF00005	tRNA	CL00001	tRNA	2.4e-12
7nww_z	1	88	RF02223	sX4			1.7e-05
1m5k_B	1	69	RF00173	Hairpin			8.6e-10
1m5k_B	1	68	RF04190	Hairpin-meta1			1.4e-07
1m5k_B	1	69	RF04191	Hairpin-meta2			3.3e-05
7nfx_1	1	299	RF00017	Metazoa_SRP	CL00003	SRP	1.7e-76
7nfx_1	1	299	RF01855	Plant_SRP	CL00003	SRP	5.1e-29
7nfx_1	17	276	RF01856	Protozoa_SRP	CL00003	SRP	1.3e-21
7nfx_1	1	297	RF01857	Archaea_SRP	CL00003	SRP	1.6e-09
7nfx_1	1	50	RF04277	mir-1268			2.4e-09
7nfx_1	14	289	RF01570	Dictyostelium_SRP	CL00003	SRP	1.1e-07
7obq_1	1	249	RF00017	Metazoa_SRP	CL00003	SRP	3.5e-58
7obq_1	1	249	RF01855	Plant_SRP	CL00003	SRP	1.3e-24
7obq_1	17	238	RF01856	Protozoa_SRP	CL00003	SRP	2.7e-21
7obq_1	1	50	RF04277	mir-1268			2e-09
7obq_1	60	238	RF01857	Archaea_SRP	CL00003	SRP	8.6e-08
7obq_1	67	231	RF01570	Dictyostelium_SRP	CL00003	SRP	6.2e-06
5aox_F	2	86	RF00017	Metazoa_SRP	CL00003	SRP	9.8e-11
5aox_F	39	1	RF03639	mir-619			2.4e-06
6mj0_B	24	104	RF00233	Tymo_tRNA-like			4.9e-16
6mj0_B	1	23	RF00390	UPSK			5.9e-07
6r6g_AF	1	206	RF00017	Metazoa_SRP	CL00003	SRP	2.2e-41
6r6g_AF	67	188	RF01856	Protozoa_SRP	CL00003	SRP	2.3e-14
6r6g_AF	1	206	RF01855	Plant_SRP	CL00003	SRP	6.6e-13
6r6g_AF	1	50	RF04277	mir-1268			1.7e-09
3jaj_4	1	206	RF00017	Metazoa_SRP	CL00003	SRP	2.7e-43
3jaj_4	1	206	RF01855	Plant_SRP	CL00003	SRP	2.6e-16
3jaj_4	17	206	RF01856	Protozoa_SRP	CL00003	SRP	9.7e-15
3jaj_4	1	50	RF04277	mir-1268			1.7e-09
3jaj_4	1	206	RF01857	Archaea_SRP	CL00003	SRP	0.00064
7sam_A	39	167	RF01084	TLS-PK3			4.2e-28
7sam_A	37	171	RF01085	TLS-PK4			8e-08
8fli_A	77	226	RF02012	group-II-D1D4-7	CL00102	group-II-D1D4	7.4e-26
8fli_A	807	884	RF00029	Intron_gpII			3.2e-09
4v5z_BE	4	51	RF03852	mir-610			7.4e-09
4v5z_BE	51	4	RF03852	mir-610			7.4e-09
4v5z_BE	1	54	RF03934	mir-m107-1			0.00038
4v5z_BE	54	1	RF03934	mir-m107-1			0.00038
6chr_A	548	621	RF00029	Intron_gpII			3e-13
6chr_A	42	199	RF02012	group-II-D1D4-7	CL00102	group-II-D1D4	1.9e-10
6chr_A	25	177	RF02003	group-II-D1D4-4	CL00102	group-II-D1D4	9.7e-05
4ue5_A	1	299	RF00017	Metazoa_SRP	CL00003	SRP	4e-76
4ue5_A	1	299	RF01855	Plant_SRP	CL00003	SRP	1.9e-28
4ue5_A	17	268	RF01856	Protozoa_SRP	CL00003	SRP	1.4e-21
4ue5_A	1	297	RF01857	Archaea_SRP	CL00003	SRP	2e-09
4ue5_A	1	50	RF04277	mir-1268			2.4e-09
4ue5_A	14	283	RF01570	Dictyostelium_SRP	CL00003	SRP	3.8e-07
4ds6_A	91	263	RF02001	group-II-D1D4-3	CL00102	group-II-D1D4	4.7e-31
4ds6_A	261	330	RF01998	group-II-D1D4-1	CL00102	group-II-D1D4	1.8e-07
4ds6_A	362	419	RF00029	Intron_gpII			8.5e-05

RNA chain	Start	End	Rfam accession	Name	Clan accesion	Clan name	E-value
6hrm_1	1,507	4,403	RF02541	LSU_rRNA_bacteria	CL00112	LSU	0.0
6hrm_1	1,506	4,411	RF02540	LSU_rRNA_archaea	CL00112	LSU	0.0
6hrm_1	1	1,528	RF00177	SSU_rRNA_bacteria	CL00111	SSU	0.0
6hrm_1	1,666	4,414	RF02543	LSU_rRNA_eukarya	CL00112	LSU	0.0
6hrm_1	6	1,460	RF01959	SSU_rRNA_archaea	CL00111	SSU	3.5e-274
6hrm_1	6	1,457	RF02542	SSU_rRNA_microsporidia	CL00111	SSU	8.3e-199
6hrm_1	6	1,522	RF01960	SSU_rRNA_eukarya	CL00111	SSU	8.4e-181
6hrm_1	1,519	1,672	RF00002	5_8S_rRNA	CL00112	LSU	2.5e-07
6hrm_1	4,372	4,445	RF00177	SSU_rRNA_bacteria	CL00111	SSU	0.00072
8dvs_B	1	64	RF04070	MIR6440			2.1e-05
8dvs_B	64	1	RF04070	MIR6440			3.1e-05
8dvs_B	3	62	RF03819	mir-Ro6-3			4.2e-05
8dvs_B	62	3	RF03819	mir-Ro6-3			4.3e-05
8dvs_B	64	1	RF00729	mir-278			5.1e-05
8dvs_B	5	60	RF03934	mir-m107-1			8e-05
8dvs_B	64	1	RF03771	mir-341			9.5e-05
8dvs_B	60	5	RF03934	mir-m107-1			0.00011
8dvs_B	1	64	RF03771	mir-341			0.00012
8dvs_B	2	63	RF04026	mir-8499			0.00023
8dvs_B	63	2	RF04026	mir-8499			0.00024
8dvs_B	62	3	RF01688	Actino-pnp			0.00037
8gzq_S	2	70	RF02340	DENV_SLA	CL00129	Flavivirus-5UTR	9.2e-18
8gzq_S	74	168	RF00185	Flavi_CRE			1.8e-09
8gzq_S	2	113	RF03546	Flavivirus-5UTR	CL00129	Flavivirus-5UTR	4.6e-06
7aor_2	2,020	2,579	RF02546	LSU_trypano_mito	CL00112	LSU	9.7e-138
7aor_2	2,589	3,210	RF02545	SSU_trypano_mito	CL00111	SSU	4.5e-120
4ujd_BC	2	354	RF00061	IRES_HCV	CL00017	IRES1	5.4e-138
4ujd_BC	128	321	RF00209	IRES_Pesti	CL00017	IRES1	2.6e-10
4ujd_BC	387	485	RF00620	HCV_ARF_SL			2.6e-06
8t2s_B	101	278	RF02001	group-II-D1D4-3	CL00102	group-II-D1D4	1.5e-28
8t2s_B	573	652	RF00029	Intron_gpII			4.1e-08
8t2s_B	315	393	RF01998	group-II-D1D4-1	CL00102	group-II-D1D4	5e-06
6wnw_4	1	3,031	RF02541	LSU_rRNA_bacteria	CL00112	LSU	0.0
6wnw_4	1	3,031	RF02540	LSU_rRNA_archaea	CL00112	LSU	0.0
6wnw_4	160	3,021	RF02543	LSU_rRNA_eukarya	CL00112	LSU	0.0
6wnw_4	1,069	1,185	RF00001	5S_rRNA	CL00113	5S_rRNA	2.2e-08
6wnw_4	13	166	RF00002	5_8S_rRNA	CL00112	LSU	1.7e-07
5lzf_x	13	48	RF01988	SECIS_2			1.2e-06
5lzf_x	15	48	RF01989	SECIS_3			6.6e-05
8s95_C	32	122	RF00386	Entero_5_CRE			8.4e-26
8s95_C	2	154	RF00005	tRNA	CL00001	tRNA	1.3e-09
8fti_B	3	67	RF04036	mir-2076			2.4e-08
8fti_B	64	2	RF00827	mir-77			9.6e-06
8fti_B	59	10	RF03658	mir-2300			1.7e-05
8fti_B	10	59	RF03658	mir-2300			1.7e-05
8fti_B	67	2	RF04294	mir-3578	CL00149	mir-154	3.6e-05
8fti_B	7	62	RF03257	mir-4427			4.8e-05
8fti_B	66	3	RF03404	mir-1298			8.4e-05
8fti_B	3	66	RF03404	mir-1298			0.00011
8fti_B	1	69	RF04295	mir-329	CL00149	mir-154	0.00012
8fti_B	11	58	RF03938	mir-4662			0.00014
8fti_B	6	65	RF03601	MIR7993			0.00019
8fti_B	1	72	RF03743	MIR7696			0.00019
8fti_B	70	2	RF00928	mir-590			0.00029
8fti_B	76	1	RF03743	MIR7696			0.0003
8fti_B	62	7	RF03257	mir-4427			0.00089
1e8s_C	21	88	RF00017	Metazoa_SRP	CL00003	SRP	1.7e-10
1e8s_C	19	70	RF04277	mir-1268			2.6e-10
1e8s_C	21	88	RF01855	Plant_SRP	CL00003	SRP	0.00012
1grz_B	1	237	RF00028	Intron_gpI			7.4e-30
1grz_B	125	246	RF03000	LOOT			0.00048
6q97_7	1	74	RF00005	tRNA	CL00001	tRNA	2.8e-15
6q97_7	77	39	RF02194	HPnc0260			2.4e-06
7aih_1	2,400	2,963	RF02546	LSU_trypano_mito	CL00112	LSU	8.6e-140
7aih_1	2,984	3,610	RF02545	SSU_trypano_mito	CL00111	SSU	3.5e-130
7lyf_A	31	100	RF02340	DENV_SLA	CL00129	Flavivirus-5UTR	6.6e-16
7lyf_A	31	139	RF03546	Flavivirus-5UTR	CL00129	Flavivirus-5UTR	8.8e-11
7lyf_A	1	136	RF00005	tRNA	CL00001	tRNA	4.6e-10

RNA chain	Start	End	Rfam accession	Name	Clan accesion	Clan name	E-value
8sp9_C	30	119	RF00386	Entero_5_CRE			4.5e-28
8sp9_C	1	150	RF00005	tRNA	CL00001	tRNA	1.1e-07
4v5z_BF	8	113	RF03296	MIR8788			3.8e-10
4v5z_BF	113	8	RF03296	MIR8788			3.8e-10
4v5z_BF	2	119	RF04104	MIR918			6e-10
4v5z_BF	119	2	RF04104	MIR918			6e-10
4v5z_BF	18	104	RF04248	MIR7486			5.4e-09
4v5z_BF	103	17	RF04248	MIR7486			5.4e-09
4v5z_BF	32	89	RF04076	mir-H11			2.8e-07
4v5z_BF	89	32	RF04076	mir-H11			2.8e-07
4v5z_BF	20	101	RF03703	mir-3622			4.8e-07
4v5z_BF	101	20	RF03703	mir-3622			4.8e-07
4v5z_BF	10	111	RF03976	MIR898			8.5e-05
4v5z_BF	111	10	RF03976	MIR898			8.5e-05
4v5z_BF	21	99	RF04162	MIR9662			0.00038
4v5z_BF	100	22	RF04162	MIR9662			0.00038
6ydp_AA	1,176	2,737	RF02541	LSU_rRNA_bacteria	CL00112	LSU	2.5e-131
6ydp_AA	1,173	2,738	RF02540	LSU_rRNA_archaea	CL00112	LSU	9.8e-92
6ydp_AA	66	1,037	RF00177	SSU_rRNA_bacteria	CL00111	SSU	1e-80
6ydp_AA	71	1,035	RF01959	SSU_rRNA_archaea	CL00111	SSU	2.2e-56
6ydp_AA	71	1,032	RF02542	SSU_rRNA_microsporidia	CL00111	SSU	3.4e-46
6ydp_AA	2,141	2,541	RF02543	LSU_rRNA_eukarya	CL00112	LSU	8e-33
6ydp_AA	1,915	2,080	RF02543	LSU_rRNA_eukarya	CL00112	LSU	5.2e-12
6ydp_AA	2,669	2,743	RF00005	tRNA	CL00001	tRNA	6.6e-10
6ydp_AA	5,333	5,268	RF00005	tRNA	CL00001	tRNA	1.2e-09
6ydp_AA	3,701	3,769	RF00005	tRNA	CL00001	tRNA	1.6e-09
6ydp_AA	3,839	3,767	RF00005	tRNA	CL00001	tRNA	1.4e-08
6ydp_AA	9,408	9,476	RF00005	tRNA	CL00001	tRNA	2.3e-07
6ydp_AA	11,561	11,629	RF00005	tRNA	CL00001	tRNA	5.6e-07
6ydp_AA	14,159	14,091	RF00005	tRNA	CL00001	tRNA	1.9e-06
6ydp_AA	5,094	5,027	RF00005	tRNA	CL00001	tRNA	1e-05
6ydp_AA	5,170	5,096	RF00005	tRNA	CL00001	tRNA	1.6e-05
6ydp_AA	1	70	RF00005	tRNA	CL00001	tRNA	1.7e-05
6ydp_AA	3,841	3,910	RF00005	tRNA	CL00001	tRNA	0.00028
6ydp_AA	6,951	6,883	RF00005	tRNA	CL00001	tRNA	0.00041
6ydp_AA	16,450	16,378	RF04217	mir-297			0.0005
7lyg_A	32	102	RF02340	DENV_SLA	CL00129	Flavivirus-5UTR	2.8e-10
7lyg_A	1	139	RF00005	tRNA	CL00001	tRNA	1e-09
1e8o_E	3	48	RF00017	Metazoa_SRP	CL00003	SRP	4.6e-09
1e8o_E	1	50	RF04277	mir-1268			2.6e-08
1e8o_E	3	48	RF01855	Plant_SRP	CL00003	SRP	0.00041
2zue_B	1	75	RF00005	tRNA	CL00001	tRNA	2.7e-15
2zue_B	1	74	RF01852	tRNA-Sec	CL00001	tRNA	8.3e-05
2zue_B	19	74	RF01684	mascRNA-menRNA			0.00021
8uw3_B	55	1	RF01940	hvt-mir-H			0.00011
8uw3_B	59	1	RF03516	MIR5229			0.00014
8uw3_B	54	1	RF03654	MIR4239			0.00032
8uw3_B	61	1	RF03872	MIR6426			0.00081
8uw3_B	72	1	RF02481	GlsR18			0.00095
3add_D	1	88	RF01852	tRNA-Sec	CL00001	tRNA	7.3e-15
3add_D	2	88	RF00005	tRNA	CL00001	tRNA	0.00018
3add_D	20	87	RF01684	mascRNA-menRNA			0.00032
5f9r_A	38	116	RF02348	tracrRNA			1e-12
5f9r_A	50	21	RF01335	CRISPR-DR22			4.8e-05
5f9r_A	37	66	RF01335	CRISPR-DR22			0.00048
6uz7_8	1	1,507	RF01960	SSU_rRNA_eukarya	CL00111	SSU	0.0
6uz7_8	1	1,507	RF02542	SSU_rRNA_microsporidia	CL00111	SSU	3.3e-238
6uz7_8	1	1,510	RF01959	SSU_rRNA_archaea	CL00111	SSU	1.6e-180
6uz7_8	1	1,512	RF00177	SSU_rRNA_bacteria	CL00111	SSU	1.2e-164
6uz7_8	2,140	2,825	RF02543	LSU_rRNA_eukarya	CL00112	LSU	4.6e-141
6uz7_8	1,724	2,825	RF02541	LSU_rRNA_bacteria	CL00112	LSU	8.6e-84
6uz7_8	1,975	2,825	RF02540	LSU_rRNA_archaea	CL00112	LSU	2.1e-71
6uz7_8	1,736	1,889	RF00002	5_8S_rRNA	CL00112	LSU	9.9e-45
4v5z_BI	5	68	RF03976	MIR898			1.1e-06
4v5z_BI	68	5	RF03976	MIR898			1.1e-06
4v5z_BI	9	65	RF04248	MIR7486			9.2e-06
4v5z_BI	64	8	RF04248	MIR7486			9.2e-06
4v5z_BI	1	72	RF03819	mir-Ro6-3			1.2e-05
4v5z_BI	72	1	RF03819	mir-Ro6-3			1.2e-05
6wkr_H	65	256	RF04159	MIR1437			9.2e-24
6wkr_H	256	65	RF04159	MIR1437			2e-23
6wkr_H	205	115	RF04162	MIR9662			1e-07
6wkr_H	116	206	RF04162	MIR9662			2.4e-07
6wkr_H	200	121	RF03691	mir-2765			4.5e-05
